# The Prognosis of IVF in Poor Responders Depending on the Bologna Criteria: A Large Sample Retrospective Study from China

**DOI:** 10.1155/2015/296173

**Published:** 2015-12-30

**Authors:** Shuo Yang, Xinna Chen, Xiumei Zhen, Haiyan Wang, Caihong Ma, Rong Li, Ping Liu, Jie Qiao

**Affiliations:** Peking University Third Hospital, No. 49, North Huayuan Road, Haidian District, Beijing 100191, China

## Abstract

*Objective*. To analyze the treatment outcomes of patients who accepted IVF/ICSI-ET, diagnosed POR according to Bologna criteria.* Study Design*. Retrospective cohort study of one reproductive medical center, from 1st Jan., 2009, to 31st Dec., 2014. All patients fulfilled the Bologna criteria and accept IVF/ICSI-ET treatment with stimulation cycle. The main outcome measures were clinical pregnancy rate (CPR) and live birth rate (LBR).* Results*. There were 5770 eligible cycles included in this study. The incidence of POR was 10.3% (6286/62194). The overall CPR was 18.7%, IR was 11.6%, LBR/ET was 11.5%, and LBR/OPU was 8.3%. The cycle cancellation for no available oocyte or embryo was 4.9% and 18.6%, respectively. The subgroup of younger POR patients got highest CPR and LBR/ET, which decreased while increasing maternal age. Within three attempts, the patients got similar CPR and LBR.* Conclusion*. In conclusion, our study supports the Bologna criteria that defined women with poor IVF outcomes. But those younger than 42 years old with the first 3 attempts of IVF could got acceptable CPR and LBR.

## 1. Introduction

The number of infertile couples is increasing year by year around the world; more and more couples rely on in vitro fertilization and embryo transfer (IVF-ET) to achieve pregnancy and get their babies. In IVF treatment, poor ovarian response (POR) to controlled ovarian stimulation (COS) is a frustrating condition that represents a topic of utmost clinical and scientific relevance [1–3]. The management of POR is still controversial. So far, lots of researches had been conducted to investigate the most suitable management for those POR patients but were still controversial. Difficulties in drawing firm conclusions are due to several reasons. Firstly, the success rate of those women was quite low, and large RCTs were hard to conduct to obtain reliable data. Secondly and most importantly, the definition of POR was quite different from different clinicians, and there were more than 40 different definitions that have been reported [4, 5]. The lack of  RCTs and the heterogeneity of the studied populations make it difficult to conduct high quality meta-analysis or get reliable conclusion.

In order to overcome the difficulties caused by the absence of a unique definition of POR, ESHRE and ASRM called for expert meeting to develop consensus on the criteria to diagnose POR, that is, the Bologna criteria, and were published in 2011 [2]. This unique consensus will promote the investigation in this field by unified research population but also needs more studies to verify the validity of the diagnose criteria. For Chinese women especially, being influenced by the traditional ideas, “donor cycle” is very rare. For those women with decreased ovarian reserve, improving the pregnancy rate of IVF will help them achieve the aspirations of becoming a mother.

Therefore, in order to evaluate the Bologna criteria in clinical practice, we conduct a retrospective study, analyzing the treatment characteristics and prognosis of women diagnosed POR depending on Bologna criteria.

## 2. Materials and Methods

### 2.1. Patient Information

A retrospective study design and data collection protocol were approved by the hospital ethics and were used to collect information on infertility couples undergoing fresh cycle of IVF/ICSI-ET treatment in unique center (Reproductive Medical Center of Peking University Third Hospital) between 1st Jan., 2009, and 31st Dec., 2014. Inclusion criteria were as follows: infertility couples underwent IVF/ICSI-ET that fulfilled the Bologna criteria [2], that is, at least two of the following three features must be present: (1) advanced maternal age (*⩾*40 years) or any other risk factor for POR; (2) a previous POR (⩽3 oocytes with a conventional stimulation protocol); and (3) an abnormal ovarian reserve test (i.e., AFC, 5–7 follicles or AMH, 0.5–1.1 ng/mL). All patients accepted stimulation cycle. The exclusion criteria included the following: (1) patients' acceptance of more than 3 cycles of IVF-ET, (2) the fact that endocrine disorders or abnormal uterus cavity could affect pregnancy rate, such as hyperprolactinemia, endometrial polyps, uterus septum or adhesion, and submucous uterine myoma, (3) nontreated hydrosalpinx, and (4) PGD cycles.

### 2.2. Hormonal Measurements

The sex hormonal assessment was performed on the second day of menstrual cycle (initiation of stimulation) and the day of HCG injection. Serum LH, FSH, E2, and P were tested using the Immulite 1000 assay based on chemiluminescence (DPC, Poway, CA). The preparation setup, dilutions, adjustment, assay, and quality control procedures were performed according to the manufacturer's instructions. The interassay coefficient of variation for all measurements was less than 10%, and the intra-assay variation was less than 15%.

### 2.3. Definition of Clinical Outcomes

The clinical pregnancy was defined by the presence of one or more intrauterine gestational sacs. The live birth was defined by given birth between 28 and 42 gestational weeks. The analysis of live birth rate has included cycles between 1st Jan., 2009, and 31st Dec., 2013.

### 2.4. Statistical Analysis

Values were expressed as mean ± standard deviation (SD) and median. The statistical analysis was performed with the Statistical Package for Social Sciences (version 13.0; SPSS, Chicago, IL). A *p* value of <0.05 was considered to be statistically significant.

## 3. Results

**Figure 1:**
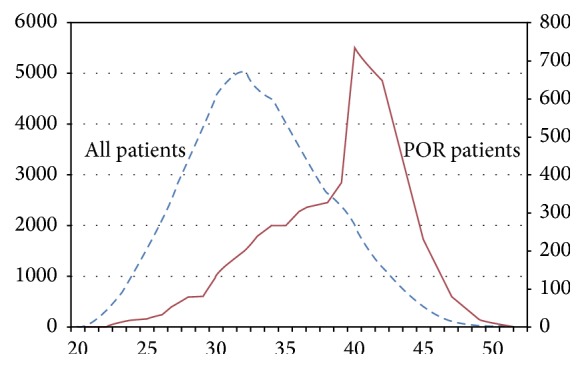
The distribution of age in all patients and POR.

There were 62,194 oocyte retrieval cycles in our center between 1st Jan., 2009, and 31st Dec., 2014, 6386 cycles meet the Bologna criteria, and the incidence of POR was 10.3% (6286/62194). According to the inclusion and exclusion criteria, 5770 cycles were included in the study. The basic characteristics of patients were shown in Table 1. The distribution of age in all patients and POR was shown in Figure 1.

The overall treatment outcomes were shown in Table 2, including clinical pregnancy (CPR) and live birth rate per embryo transfer cycle and oocyte pick-up cycle (LBR per ET and LBR per OPU), the cycle of no available oocyte or embryo. The overall CPR was 18.7% and LBR per embryo transfer was 11.5%. The overall cycle cancellation rate for no available oocyte or embryo was 4.9% and 18.6%, respectively.

The outcomes of patients with different treatment cycles were listed in Table 3. Within three attempts, the patients got similar CPR. Although the patients with first IVF-ET attempts got the highest implantation rate (IPR), the LBR per ET and OPU and cycle cancellation rate for no available oocyte or embryo were similar.

**Figure 2:**
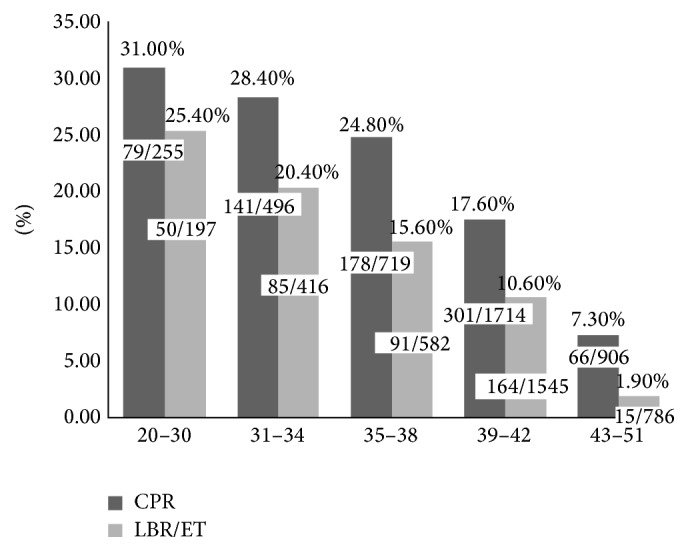
CPR and LBR/ET in women of different ages.

Depending on the Bologna criteria, patients who meet two of three criteria can be diagnosed as POR. The patients were divided into four subgroups depending on the diagnostic criteria, and the basic characteristics and clinical outcomes were listed in Table 4. And as age is an important factor affecting ovarian reserve and IVF outcomes, we further analyzed the clinical outcomes in women of different ages (Figure 2). Even being diagnosed with POR, the younger subgroup (OOCYTE + AFC) got significantly higher CPR, IPR, and LBR. In the subgroup of patients diagnosed POR by age and AFC, despite getting significantly more oocytes, still got lower CPR, IPR, and LBR than those in younger subgroup. And the subgroup of patients who fulfilled all three criteria or advanced age with 3 or less oocytes got significantly lower CPR and LBR. The CPR and LBR/ET in women of different ages also showed a decreasing successful rate with increasing ages.

## 4. Discussion

In 1983, Garcia et al. first defined poor responder (case report) [6]. Loumaye first reported that the cancellation rate of POR was 20% [7]. POR made it difficult to get enough high quality oocytes, and with poor clinical prognosis. There are lots of publications focusing on POR, but until 2011, there was no unique definition of POR.

In 2011, Ferraretti et al. first gave standard definition of POR, that is, Bologna Criteria (ESHRE and ASRM Criteria, 2011) [2]. The aim of this study is to analyze the treatment outcomes of women diagnosed POR by Bologna criteria. As far as we know, this is the largest retrospective study focusing on the treatment outcomes for those diagnosed POR according to Bologna criteria. In the present study population, the incidence of POR was 10.3%. The mean age was over 38, basal FSH was higher than 9 mIU/mL, and AFC was less than 5, all of above indicating a decreased ovarian reserve and poor treatment outcomes. The overall CPR was 18.7%, IPR was 11.6%, LBR per ET cycle was 11.5%, and 23.6% cycles were cancelled for no available oocyte or embryo. Busnelli et al. reported a similar outcome in a retrospective study with LBR was 6% [8], while Joyce Chai et al. reported a higher LBR (23.8%) [9]. So we can conclude that the Bologna criteria define a population with a low rate of success.

Although all patients were diagnosed POR by Bologna criteria, further analysis still showed a decreased success rate with increasing maternal age. Particularly for those under 38 years old, they also got acceptable CPR and LBR; even for those of 39–42 years, the CPR and LBR were 17.6% and 10.6%, respectively. For the four subgroups of POR patients, the younger patients (diagnosed POR by oocytes retrieved and AFC) also got better outcomes (CPR 25.7%, LBR 17.8%). So we conclude that even though the patients diagnosed POR, some of them still can get acceptable outcomes, especially for those under 42 years old. This conclusion is quite important for Chinese women because of the rare opportunity of donor cycle and for those who want their own biological baby.

Another challenging question for POR patients is when to stop treatment. Patrizio et al. conducted a worldwide survey, including 196 centers. To the question, “assuming no financial constraints, is there a maximum number of failed cycles after which you recommend stopping?” 39% of the respondent-cycles reported that they would stop after two failed cycles; 34% after three failed cycles; 13% after four failed cycles; and 7% after five failed cycles, and 7% had no limit on the number of failed cycles [10]. In the present study, patients got similar CPR and LBR within three attempts. So we conclude that, assuming no financial constraints, at least 3 attempts could provide for POR patients.

The COS protocol is another important factor affecting success rate of IVF treatment. The most suitable COS protocol for POR is still controversial; quite some clinicians support the fact that the mild stimulation protocols may have a more definitive role in poor responders in the future [10]. The present study was a retrospective study and did not compare the outcomes between different COS protocols. Further study is still needed to investigate the most suitable COS protocol to improve IVF outcomes.

## 5. Conclusion

In conclusion, our study supports the Bologna criteria that defined women with poor IVF outcomes. But those younger than 42 years old with the first 3 attempts of IVF could got acceptable CPR and LBR. Further study is still needed to investigate the most suitable COS protocol to improve IVF outcomes.

## Figures and Tables

**Table 1:** The basic characteristics of all patients.

	Mean ± SD	Median
AGE (yr)	38.8 ± 4.8	40
Duration of infertility (yr)	7.1 ± 5.5	5
BMI (kg/m^2^)	22.7 ± 3.2	22.3
Basal FSH (mIU/mL)	9.9 ± 5.2	9.2
AFC	4.3 ± 2.0	4
*E*_2_ on hCG day (pmol/L)	4148.0 ± 3785.9	3018
Oocytes retrieved	3.3 ± 3	3
Gn day	11 ± 3.4	11
Gn dose (IU)	3949.9 ± 1864.7	3825
Primary infertility (%)	41.4% (2388/5770)	

**Table 2:** The treatment outcomes of all patients.

	(%/*N*)
CPR	18.7/(765/4090)
IPR	11.6/(866/7444)
LBR (ET)	11.5/(405/3526)
LBR (OPU)	8.3/(405/4886)
No available oocyte	4.9/(285/5770)
No available embryo	18.6/(1075/5770)

**Table 3:** The outcome of patients with different treatment cycles.

Cycle Number	1	2	3	*p*
Number (%/*N*)	57.2 (3299/5770)	33.5 (1933/5770)	9.3 (538/5770)	—
CPR (%/*N*)	19.8 (462/2339)	17.1 (238/1395)	18.3 (65/356)	0.122
IR (%/*N*)	12.5 (525/4193)	10.3 (269/2603)	11.1 (72/648)	0.022^a^
LBR (ET) (%/*N*)	8.9 (249/2785)	7.6 (128/1690)	6.8 (28/411)	0.144
LBR (OPU) (%/*N*)	12.4 (249/2010)	10.4 (128/1232)	9.9 (28/284)	0.148
No available oocyte (%/*N*)	4.9 (162/3299)	4.9 (94/1933)	5.4 (29/538)	0.877
No available embryo (%/*N*)	19.4 (639/3299)	17.2 (333/1933)	19.1 (103/538)	0.148

Values are % and *n*/total.

^a^Significant difference between three groups (*p* < 0.05).

**Table 4:** The basic characteristics and clinical outcomes of subgroup patients diagnosed by Bologna criteria.

	All criteria	Age + oocyte	Age + AFC	Oocyte + AFC	*p*
*N*	1772	404	1410	2184	—
AGE (yr)	41.0 ± 4.2	41.3 ± 3.9	42.0 ± 1.8	34.6 ± 3.5	0.000^g^
Duration of infertility (yr)	7.6 ± 6.2	7.4 ± 5.9	8.0 ± 6.0	6.1 ± 4.1	0.000^d,f,g^
Primary infertility (%)	36.5 (646/1772)	37.9 (153/404)	29.6 (418/1410)	53.6 (1171/2184)	0.000^a^
BMI (kg/m^2^)	22.8 ± 3.1	22.7 ± 3.3	23.0 ± 3.0	22.3 ± 3.2	0.000^b,d,f,g^
Basal FSH (mIU/mL)	10.2 ± 5.4	9.5 ± 5.4	10.3 ± 4.3	9.9 ± 5.3	0.035^b,e^
AFC	3.7 ± 1.7	9.6 ± 2.5	4.7 ± 1.8	4.2 ± 1.8	0.000^a^
*E*_2_ on hCG day (pmol/L)	2847.8 ± 2390.5	2836.1 ± 2390.1	7564.1 ± 4899.6	3213.8 ± 2537.7	0.000^g^
Oocytes retrieved	2.7 ± 1.6	4.3 ± 4.3	7.1 ± 3.7	2.9 ± 1.8	0.000^a^
Gn day	10.9 ± 3.7	10.8 ± 4.2	11.3 ± 2.7	11.1 ± 3.5	0.000^a^
Gn dose (IU)	3821.3 ± 2031.4	3771.1 ± 2016.6	4293.4 ± 1517.6	3862.0 ± 1873.8	0.002^c,e,g^
ICSI (%)	33.1 (587/1772)	35.4 (143/404)	40.1 (565/1401)	31.5 (687/2184)	0.000^c,g^
MII (%)	85.5 (1088/1272)	83.5 (256/310)	79.5 (3406/4282)	85.8 (1343/1565)	0.000^c,g^
ET	1.5 ± 0.6	1.5 ± 0.6	2.5 ± 0.7	1.5 ± 0.6	0.000^c,e,g^
CPR (%)	13.1 (148/1128)	16.0 (40/250)	16.3 (208/1274)	25.7 (369/1438)	0.000^d,f,g^
IPR (%)	9.4 (158/1681)	11.9 (45/377)	10.6 (340/3206)	19.4 (423/2180)	0.000^b,d,f,g^
LBR per ET (%)	7.7 (75/974)	6.3 (11/176)	9.2 (111/1209)	17.8 (208/1167)	0.000^b,d,f,g^
LBR per OPU (%)	5.0 (75/1493)	3.8 (11/290)	8.3 (111/1331)	11.7 (208/1772)	0.000^b,d,f,g^
No available oocyte (%)	6.8 (121/1772)	9.4 (38/404)	0	5.8 (126/2184)	0.000^e,f,g^
No available embryo (%)	23.5 (416/1772)	22.5 (91/404)	4.5 (63/1410)	23.1 (505/2184)	0.000^c,e,g^

Values are % and *n*/total or mean ± SD

^a^Significant difference between four groups (*p* < 0.05).

^b^Significant difference between all and age + oocyte (*p* < 0.05).

^c^Significant difference between all and age + AFC (*p* < 0.05).

^d^Significant difference between all and oocyte + AFC (*p* < 0.05).

^e^Significant difference between age + oocyte and AGE + AFC (*p* < 0.05).

^f^Significant difference between age + oocyte and oocyte + AFC (*p* < 0.05).

^g^Significant difference between age + AFC and oocyte + AFC (*p* < 0.05).
